# Synthesis and Characterisation of Novel Tricyclic and Tetracyclic Furoindoles: Biological Evaluation as SAHA Enhancer against Neuroblastoma and Breast Cancer Cells

**DOI:** 10.3390/molecules26195745

**Published:** 2021-09-22

**Authors:** Murat Bingul, Greg M. Arndt, Glenn M. Marshall, David StC. Black, Belamy B. Cheung, Naresh Kumar

**Affiliations:** 1 School of Chemistry, The University of New South Wales, Sydney, NSW 2052, Australia; muratbingul1983@gmail.com; 2 Lowy Cancer Research Centre, Children’s Cancer Institute, UNSW Sydney, Sydney, NSW 2031, Australia; GArndt@ccia.unsw.edu.au (G.M.A.); gmarshall@ccia.unsw.edu.au (G.M.M.); 3 School of Pharmacy, Dicle University, Diyarbakır 21280, Turkey; 4 ACRF Drug Discovery Centre for Childhood Cancer, Children’s Cancer Institute Australia for Medical Research, Lowy Cancer Research Centre, UNSW Sydney, Sydney, NSW 2052, Australia; 5 Kids Cancer Centre, Sydney Children’s Hospital, Randwick, NSW 2031, Australia; 6 School of Women’s and Children’s Health, UNSW Sydney, Sydney, NSW 2052, Australia

**Keywords:** furoindole, SAHA enhancement, HDAC inhibitors, neuroblastoma, breast cancer

## Abstract

The dihydropyranoindole structures were previously identified as promising scaffolds for improving the anti-cancer activity of histone deacetylase inhibitors. This work describes the synthesis of related furoindoles and their ability to synergize with suberoylanilide hydroxamic acid (SAHA) against neuroblastoma and breast cancer cells. The nucleophilic substitution of hydroxyindole methyl esters with α-haloketones yielded the corresponding arylether ketones, which were subsequently cyclized to tricyclic and tetracyclic furoindoles. The furoindoles showed promising individual cytotoxic efficiency against breast cancer cells, as well as decent SAHA enhancement against cancer cells in select cases. Interestingly, the best IC_50_ value was obtained with the non-cyclized intermediate.

## 1. Introduction

Suberoylanilide hydroxamic acid (SAHA, also known as vorinostat) is an anticancer therapeutic agent due to its histone deacetylase (HDAC) inhibitory activity [1,2,3]. The inhibition of HDAC enzymes results in the accumulation of acetylated histones, which in turn leads to an increase in transcriptionally active chromatin [4,5]. HDAC inhibitors have been reported to suppress cell proliferation and angiogenesis, induce cell differentiation, and promote apoptosis in a number of cancer cell types [6,7]. Although SAHA has been approved for the treatment of cutaneous T-cell lymphoma as a monotherapy, it is still susceptible to resistance arising from mutation of cancer cells [8]. Moreover, as SAHA targets only one signalling pathway or molecular mechanism, single-agent treatment with SAHA was ineffective against several cancers [9,10]. Hence, the combination of SAHA with other chemotherapeutic agents with different mechanisms of action has been considered to be a promising approach to overcome resistance to single-agent therapies [9,10,11].

The starting point for this study was the screening of 10,560 compounds from the Walter and Eliza Hall Institute (WEHI) library to identify molecules that could act synergistically with SAHA to overcome drug resistance in SAHA-resistant MDA-MB-231 breast cancer cell lines [12]. By considering the structural features of the hit compounds, briefly tricyclic or tetracyclic nitrogen containing aromatic compounds, our previous study [13] investigated related heterocyclic molecules for activity against SH-SY5Y neuroblastoma and MDA-MB-231 breast cancer cell lines [14]. It was concluded that indole systems, and dihydropyranoindole derivatives in particular, were viable lead structures for the development of novel SAHA enhancers with low toxicity to normal cells.

The previous work also suggested that furo moieties could be promising targets for SAHA enhancers [13]. The main work in this manuscript describes the synthesis, characterization and in vitro biological evaluation of a series of tricyclic and tetracyclic furoindole derivatives. The effectiveness of the novel compounds as single agents and in combination with a clinical dose of SAHA was determined against neuroblastoma and breast cancer cells.

## 2. Results and Discussion

### 2.1. Chemistry

#### 2.1.1. Preparation of Hydroxy-Indole Methyl-2-Carboxylates

**Scheme 1:**
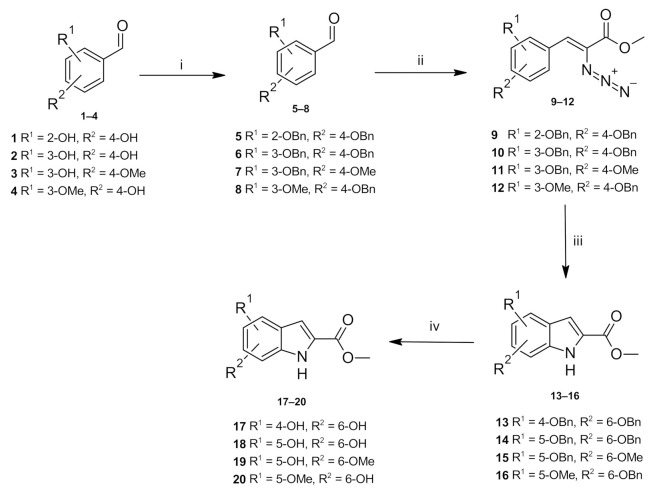
*Reagents and conditions*: (i) benzyl bromide, K_2_CO_3_, DMF, reflux, overnight; (ii) methyl azidoacetate, NaOMe, anhydrous MeOH, <10 °C, 4 h; (iii) xylene, reflux, 2 h, (iv) 5% Pd/C, H_2_, MeOH/THF, 2 h, rt.

In the current work, the targeted indoles were synthesized by the Hemetsberger indole synthesis [15] (Scheme 1). First, the substituted benzaldehydes **1**–**4** were reacted with benzyl bromide in the presence of potassium carbonate in acetone to afford the protected carbaldehydes **5**–**8** [16,17,18,19], respectively. The benzyl-protected carbaldehydes were condensed with methyl azidoacetate in the presence of sodium methoxide to obtain the azidoacrylate intermediates **9**–**12** [16,20,21]. Thermal decomposition of the arylazido esters was performed by heating at reflux in xylene, generating the methyl indole-2-carboxylates **13**–**16** in good yields. Hydrogenolysis of the benzyl group was carried out by treating the compounds with 5% *w*/*w* palladium on carbon under a hydrogen atmosphere at room temperature, yielding the desired methyl 4,6-dihydroxyindole-2-carboxylate **17** [22], methyl 5,6-dihydroxyindole-2-carboxylate **18**, methyl 5-hydroxy-6-methoxyindole-3-carboxylate **19** [23] and methyl 6-hydroxy-5-methoxyindole-2-carboxylate **20** [24].

The ^1^H-NMR spectra of methyl 5-hydroxy-6-methoxyindole-2-carboxylate **19** and methyl 6-hydroxy-5-methoxyindole-2-carboxylate **20** in CDCl_3_ contained an identical number of signals differing in their splitting patterns and the resulting coupling constants. In the spectrum of compound **19**, the H4 proton was observed as a doublet at 6.86 ppm, due to the coupling to neighbouring OH proton with a 0.9 Hz coupling constant. Furthermore, the H7 proton was observed as a singlet at 7.16 ppm. In the case of the compound **20**, H7 resonated as a doublet at 6.96 ppm with a 0.9 Hz coupling constant and H4 resonated as a singlet at 7.06 ppm. The OH proton resonated as a broad singlet at 5.57 and 5.99 ppm in the spectra of compounds **19** and **20**, respectively. Additionally, in the spectra of compounds **19** and **20**, respectively, H3 was observed as doublets at 7.11 and 7.06 ppm (*J* = 2.1 Hz for both indoles) due to the coupling with the NH proton, while the NH group was observed at 8.77 and 8.86 ppm, in the spectra of compounds **19** and **20**, respectively. The obtained ^1^H-NMR data are consistent with the reference data [23,24].

The ^1^H-NMR spectrum of methyl 4,6-dihydroxyindole-2-carboxylate **17** in *d*_6_-DMSO showed two broad singlet resonances at 9.17 and 9.66 ppm corresponding to the hydroxyl protons. The H5 and H7 resonances appeared as doublets of doublets signals at 6.00 and 6.26 ppm, respectively, with 2.3 and 0.9 Hz coupling constants arising from *meta* coupling to each other as well as coupling with the hydroxy groups. Similarly, in the ^1^H-NMR spectrum of methyl 5,6-dihydroxyindole-2-carboxylate **18** in *d*_6_-DMSO, the hydroxy protons were observed as broad singlets at 8.84 and 9.17 ppm, while the H4 and H7 protons resonated as doublets at 6.79 and 6.90 ppm with a coupling constant of 0.8 Hz due to coupling with the nearby hydroxy groups. The followed synthetic strategy was applied for the first time to produce the compound **18**.

#### 2.1.2. Synthesis of Furo [2,3-G]indoles and Furo [3,2-E]indoles via Methyl 5-Hydroxy-6-Methoxy and 6-Hydroxy-5-Methoxyindole-2-Carboxylates

**Scheme 2:**
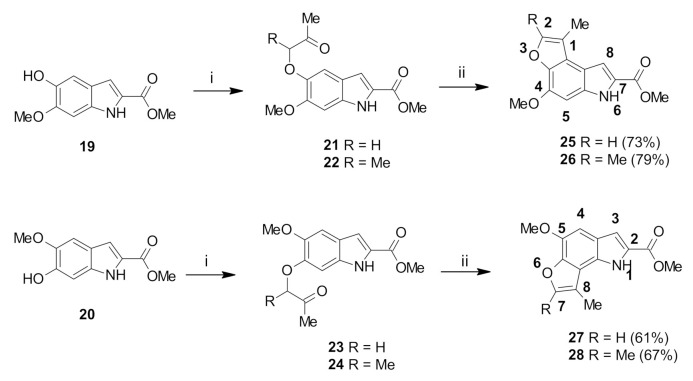
*Reagents and conditions*: (i): chloroacetone or 3-chloro-2-butanone, acetone, K_2_CO_3_, reflux, overnight; **21** (81%), **22** (85%), **23** (78%) and **24** (81%) (ii): DCM, TFA, reflux overnight.

Following the successful preparation of the indole precursors, the tricyclic furoindole systems were targeted. Methyl 5-hydroxy-6-methoxyindole-3-carboxylate **19** and methyl 6-hydroxy-5-methoxyindole-2-carboxylate **20** were refluxed with chloroacetone in acetone in the presence of potassium carbonate to afford intermediates **21** and **23**, respectively (Scheme 2). Intermediates **22** and **24** were prepared in a similar manner using 3-chloro-2-butanone, and were obtained.

The ^1^H-NMR spectra of compounds **21** and **23** displayed singlet resonances corresponding to the α-CH_2_ protons at 4.65 and 4.64 ppm, respectively. For **21**, H7 and H4 were observed as doublets at 6.91 and 7.11 ppm (*J* = 0.9 Hz), respectively, while for **23**, H4 and H7 appeared as two doublet signals at 6.80 and 7.13 ppm (*J* = 0.7 Hz), respectively. Peaks corresponding to the COCH_3_ group were observed as singlets at 2.34 and 2.33 ppm for intermediates **21** and **23**, respectively. Further confirmation of the presence of the methylene group was provided by negative peaks at 74.9 and 74.5 ppm in the DEPT-135 spectra of compounds **21** and **23**, respectively. The ^1^H-NMR spectra of the intermediates **22** and **24** displayed similar signals in the aromatic region and for the COCH_3_ group. However, the α-CH proton appeared as quartets at 4.57 and 4.63 ppm (*J* = 6.9 Hz) for compounds **22** and **24,** respectively, while additional doublets at 1.56 and 1.58 ppm corresponding to the CH-CH_3_ protons were also observed for **22** and **24**, respectively.

Cyclization was performed by heating the intermediates **21**–**24** in DCM, using catalytic amounts of TFA. This afforded the novel tricyclic furo [2,3-*g*]indoles **25**, **26** and furo [3,2-*e*]indoles **27**, **28** (Scheme 2). Cyclization was confirmed by comparison of the ^1^H-NMR spectra of the cyclic molecules **25**–**28** with the acyclic precursors **21**–**24**. Cyclization of **21–24** to **25–28** was confirmed by the disappearance of H4 peak (for 25 and 26) and of H7 peak (for 27 and 28). Besides, singlet peak for α-CH2 in 21 and 23 disappeared and were replaced by a new doublet signal at 7.51 and 7.49 ppm in the cyclized products **25** and **27**, respectively. The splitting of these peaks was rationalized as being due to coupling between the H2 proton and the C1 methyl group on the furan ring. Cyclization of **22** and **24** was analogously demonstrated by the disappearance of the α-CH multiplet in the spectra of the corresponding products **26** and **28**.

#### 2.1.3. Synthesis of Difuro [2,3-e:2′, 3′-G]indoles via Methyl 4,6-Dihydroxyindole-2-Carboxylate

**Scheme 3:**
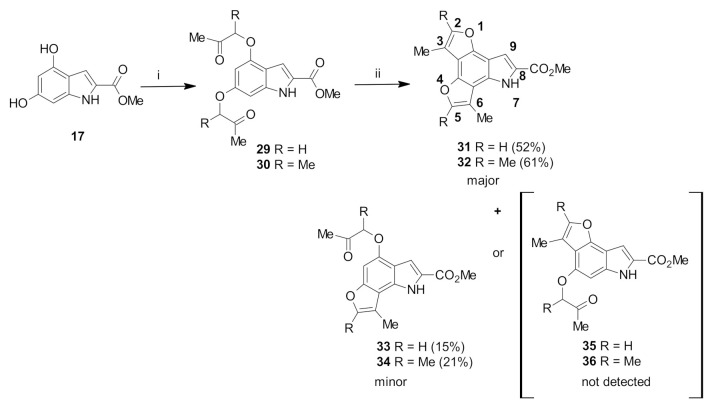
*Reagents and conditions*: (i): chloroacetone or 3-chloro-2-butanone, acetone, K_2_CO_3_, reflux overnight, **29** (82%) and **30** (88%); (ii): DCM, TFA, reflux overnight.

Due to the successful preparation of the tricyclic furoindole system, it was envisaged that this synthetic route would be applicable to the preparation of furoindole systems [25]. The interesting tetracyclic furoindoles were attempted to be synthesized via the treatment of methyl dihydroxyindole-2-carboxylate **17** with two equivalents of α-haloketones. The nucleophilic substitution reaction of methyl 4,6-dihydroxyindole-2-carboxylate **17** with chloroacetone proceeded rapidly in the presence of potassium carbonate to form the dialkoxy derivative intermediate **29** in 82% yield (Scheme 3). Similarly, when compound **17** was condensed with 3-chloro-2-butanone under the same conditions, the desired intermediate **30** was formed as a white solid in an excellent yield of 88%.

The ^1^H-NMR spectrum of intermediate **29** showed the presence of the α-CH_2_ protons as singlets at 4.59 and 4.67 ppm, together with two singlets at 2.33 pm and 2.39 ppm corresponding to the COCH_3_ protons. H5 and H7 appeared as doublet at 6.28 ppm and 6.37 ppm, respectively (*J* = 1.7 Hz), due to meta coupling with each other. Meanwhile, the most significant feature in the ^1^H-NMR spectrum of compound **30** were two quartet signals at 4.55 and 4.78 ppm, corresponding to the two α-CH protons, with *J* = 6.9 Hz due to coupling to the neighbouring CH_3_ protons. The CH-CH_3_ protons appeared as doublets at 1.52 ppm and 1.59 ppm, with the same coupling constant. Two singlet signals were observed at 2.16 and 2.24 ppm, which corresponded to the COCH_3_ protons.

When the acid-catalysed cyclization of ethers **29** and **30** was attempted, the reactions yielded two products (Scheme 3). After separation of the product mixtures by chromatography, the major products were identified as the desired tetracyclic compounds **31** and **32**. The minor products appeared more polar than **31** and **32** by TLC analysis, so they were presumed to be the products arising from only one cyclization reaction. Spectroscopic analysis showed that the C7-cyclized compounds **33** and **34** had formed, rather than the C5-cyclized compounds **35** and **36**.

The formation of the desired tetracyclic system **31** was confirmed by comparison of the ^1^H-NMR spectrum with that of the intermediate **29**. The absence of the doublet signals corresponding to H5 and H7 showed that cyclization reactions occurred at both positions on the benzene ring. Additional evidence was provided by the presence of doublets at 7.43 pm and 7.48 ppm, corresponding to H5 and H2 of the new polycyclic systems. Similarly, in the ^1^H-NMR spectrum of **32**, the cyclization of both methyl ketones was confirmed by the absence of the two quartet signals assigned to the two α-CH protons as well as the two doublet signals corresponding to H5 and H7. The H9 proton, appearing as a doublet due to the coupling with the neighbouring NH proton, appeared at 9.09 ppm (Scheme 3).

Monocyclization was confirmed by ^13^C-NMR spectroscopy, which displayed only one characteristic methyl ketone carbon signal at 207.9 and 210.6 ppm in the spectra of compounds **33** and **34**, respectively. Similarly, the ^1^H-NMR spectrum of **33** showed only one singlet at 4.69 ppm, assigned to the α-CH_2_ protons, and the spectrum for **30** showed only one quartet at 4.77 ppm, assigned to the α-CH proton. It was also observed that one aromatic proton (H7) on the indole benzene ring was still present, appearing as a singlet signal due to the disappearance of *meta* coupling with the other aromatic proton (H5).

**Figure 1:**
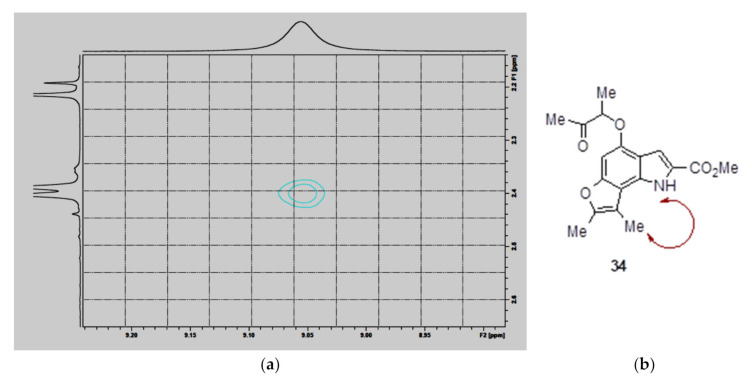
H-H NOESY NMR spectrum of compound **34** obtained by mono-cyclization of **30**.

Determining the regiochemistry of cyclization required 2D NMR spectroscopic analysis. In compound **34** (b), the H-H NOESY spectrum (a) showed a through-space correlation between the NH group (9.05 ppm) with one of the two methyl groups on the furan ring (2.40 ppm) (showed in Figure 1b). This interaction would not be possible if cyclization had occurred at C5 instead. A similar 2D NMR pattern was observed in the case of the minor product formed from **29**, thus confirming its actual structure to be the C7-cyclized compound **33**. These results are consistent with previous findings that C7 is the most reactive position in methyl 4,6-disubstituted-2-carboxylates [26].

#### 2.1.4. Attempted Synthesis of Difuro [3,2-e:2′, 3′-G]indoles with a Different Heterocycles Orien-Tation

**Scheme 4:**
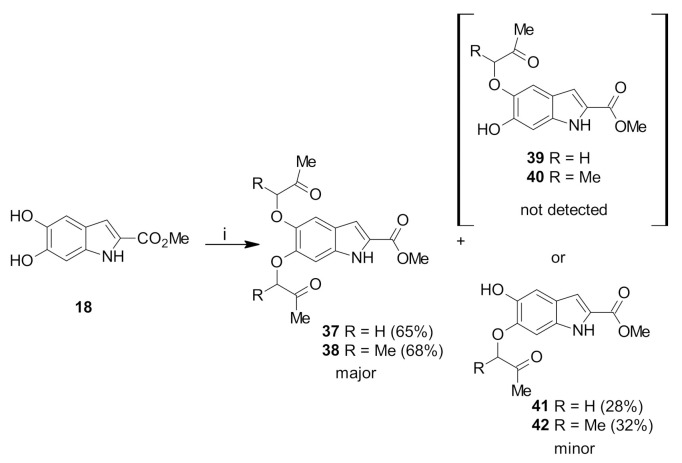
*Reagents and conditions*: (i): chloroacetone or 3-chloro-2-butanone, acetone, K_2_CO_3_, reflux overnight.

In order to prepare difuroindoles with different heterocyclic orientations, the previously developed strategy was applied to the 5,6-dihydroxyindole **18** core. The reaction of **18** with two equivalents of α-haloketones (chloroacetone and 3-chloro-2-butanone) in the presence of K_2_CO_3_ generated two major products as the di-alkylated intermediates **37** and **38** as testified by the disappearing of both the OH signals (Scheme 4), while two singlets corresponding to the α-CH_2_ protons at 4.59 and 4.67 ppm and an overlapping singlet corresponding to the two CH_3_ groups at 2.36 ppm appeared. The presence of methylene peaks at 74.4 and 74.9 ppm in the DEPT-135 spectrum further confirmed the structure of **37**. In the case of **38**, multiplet signals in the range of 1.51–1.57 ppm, corresponding to the two CH-CH_3_ protons, and multiplet signals between 4.60 and 4.69 ppm, assigned to the α-CH protons, confirmed the double alkylation of the dihydroxyindole core.

The minor products were the O-C6 mono-alkylated intermediates **41** and **42**. The ^1^H-NMR spectra of the mono-alkylated products in CDCl_3_ showed broad singlets at 5.26 and 5.61 ppm, indicating the presence of a hydroxyl group in each molecule. The characteristic singlet of the α-CH_2_ protons at 4.67 ppm and the multiplet of the α-CH proton in the range of 4.02 to 4.09 ppm matched the integrations expected for the mono-alkylated products. However, ^1^H-NMR spectroscopy was unable to assign the regiochemistry of the reaction. Instead, X-ray crystallography of a product obtained from a further stage (see below) established that HO-C6 substitution had occurred to give **41** and **42**. It is proposed that in the nucleophilic substitution reaction C6 anion is formed more easily as a result of stabilization by the conjugated COOMe group.

Attempts to cyclize the intermediate ethers **37** and **38** in the same manner as for **29** and **30** using a catalytic amount of trifluoroacetic acid in dichloromethane resulted in the recovery of unconsumed starting materials (Scheme 4). Cyclization under more strongly acidic conditions, using a 1:1 ratio of solvent mixture (TFA and DCM) and neat acid, resulted in decomposition and the formation of polymerized products. Similar results were observed for the mono-alkylated intermediate **41**, with a catalytic amount of trifluoroacetic acid giving unreacted starting materials, while more acidic conditions resulted in its decomposition. It is assumed that, while the 4,6-dihydroxy scaffold strongly increases the nucleophilicity of the C5 and C7 through resonance contribution and the cyclization occurs to produce the tetracyclic system. In the case of 5,6-dihydroxy system, the hydroxyl groups interfere with each other preventing the cyclization.

**Scheme 5:**
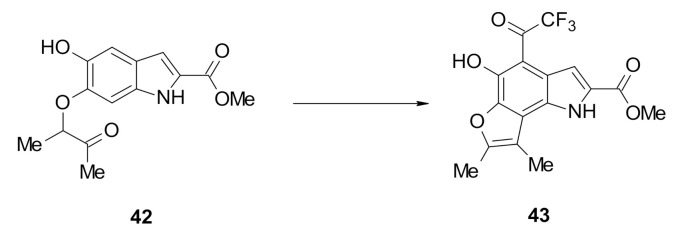
*Reagents and conditions*: DCM, TFA, reflux overnight **43** (64%).

With mono-alkylated intermediate **42**, cyclization using catalytic trifluoroacetic acid also led to no reaction. However, treatment of **42** using a 1:1 mixture of TFA and DCM afforded a single product **43** with an intense yellow colour in 64% yield (Scheme 5). The ^13^C-NMR spectrum of the product **43** showed the disappearance of the ketone carbonyl signal at 205.3 ppm, suggesting that cyclization had occurred. However, the appearance of only one aromatic proton signal (the indole H3) in the ^1^H-NMR spectrum, as well as the downfield shift of the single hydroxyl proton signal to 13.44 ppm, possibly due to strong hydrogen bonding, suggested that a substitution reaction had also occurred at the C4 position.

**Figure 2:**
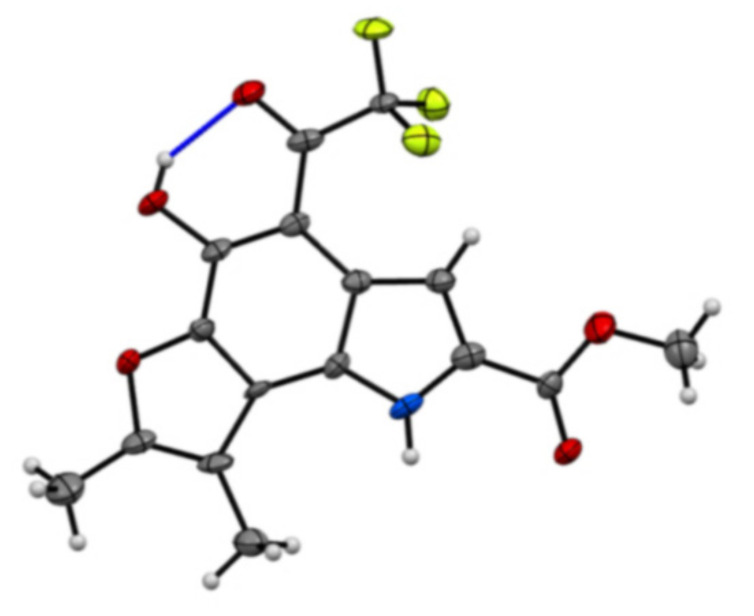
ORTEP diagram of compound **43**.

The exact structure of compound **43** was revealed by X-ray crystallographic analysis. It showed that an electrophilic substitution reaction had occurred at the C4 position with trifluoroacetic acid to generate the trifluoroacetyl-substituted furoindole. The crystal structure also revealed the presence of a hydrogen bond between the C5 hydroxyl proton and C4 trifluoroacetyl carbonyl oxygen atom, which would account for the downfield shift of the OH signal in the ^1^H-NMR spectrum of **43** (Figure 2). The yellow colour in the Figure 2 demonstrates the F atoms. The red colour indicates the O atoms and blue line proves the hydrogen bonds occurring between the hydroxyl group and the carbonyl oxygen located at the 4-position. 

In summary, the C6-hydroxy group in the 5,6-dihydroxyindole scaffold **18** was more acidic than the C5-hydroxy group, leading to the generation of the O-C6-alkylated indole. Both the mono-alkylated and di-alkylated intermediates were unreactive to cyclization using catalytic TFA, while more strongly acidic conditions led to decomposition. The only exception was methyl 5-methoxy-6-hydroxyindole-2-carboxylate **42**, which could be cyclized at C7 with concomitant substitution of a trifluoroacetyl group at C4 by using a 1:1 mixture of TFA and DCM, giving tricyclic compound **43** whose structure was confirmed by X-ray crystallographic analysis.

### 2.2. Biological Results

#### 2.2.1. Preliminary Biological Screening of Furoindoles for SAHA Enhancement Activity

**Figure 3:**
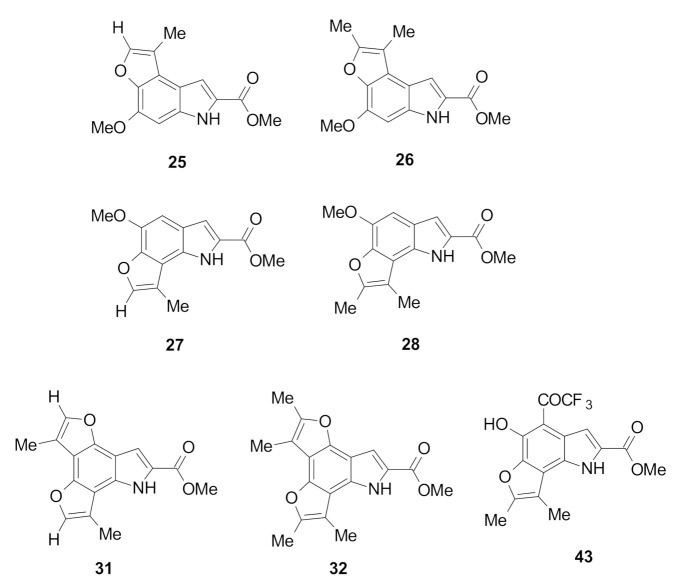
Tested furoindoles analogues against the panel of cancer cells.

The ability of selected furoindoles (Figure 3) to enhance the activity of SAHA against the SH-SY5Y and Kelly neuroblastoma and MDA-MB-231 and MCF-7 breast adenocarcinoma cell lines was examined using the Alamar blue (Resazurin) assay [14].

**Figure 4:**
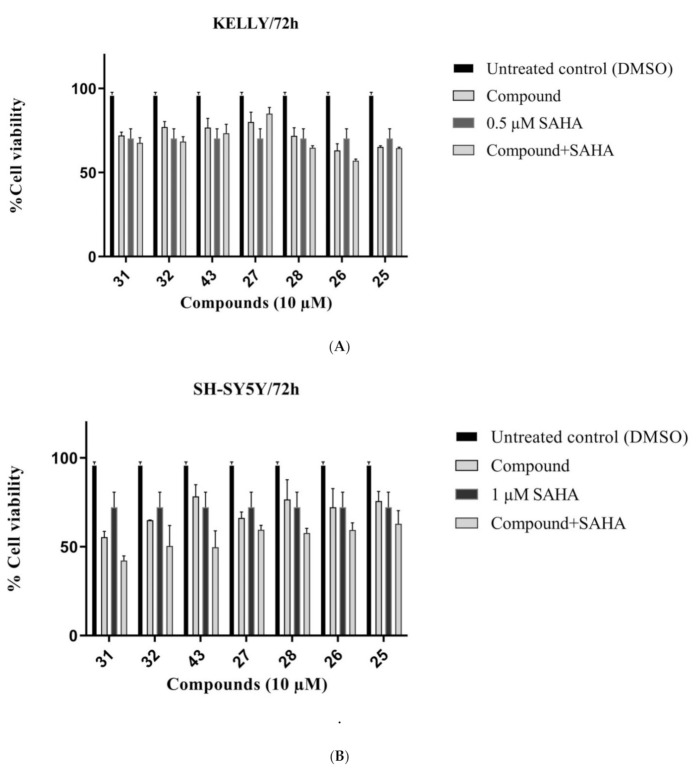
Cell viability of (**A**) KELLY (**B**) SH-SY5Y neuroblastoma cancer cells treated with 10 μM compounds over 72 h, in the presence or absence of 1 μM of SAHA. Error bars represent mean values (±S.D.) from three independent determinations.

Cell lines were treated with 1 μM SAHA (SH-SY5Y) and 0.5 μM SAHA (Kelly), 10 μM of the compounds, or their combination over 72 h, and cell viabilities were normalized to a DMSO control. The furoindole compounds generally showed moderate cytotoxic activity against both neuroblastoma cell lines. The furoindoles **26** and **25** were the most cytotoxic against Kelly cells with 37% and 36% reduction in cell viability versus DMSO (Figure 4A). However, the compounds did not potentiate the cytotoxic effect of SAHA against Kelly cells. In the case of SH-SY5Y cells, compounds **31** and **32** were the most potent with 45% and 35% inhibition of cell viability, respectively, versus DMSO (Figure 4B). Importantly, all the furoindoles enhanced the SAHA activity towards the SH-SY5Y cells with reductions in cell viability values ranging from 7–29%. The greatest SAHA enhancement was achieved by compound **43**, where the combination decreased SH-SY5Y cell viability (41%) by a further 29% compared to SAHA alone (70%).

**Figure 5:**
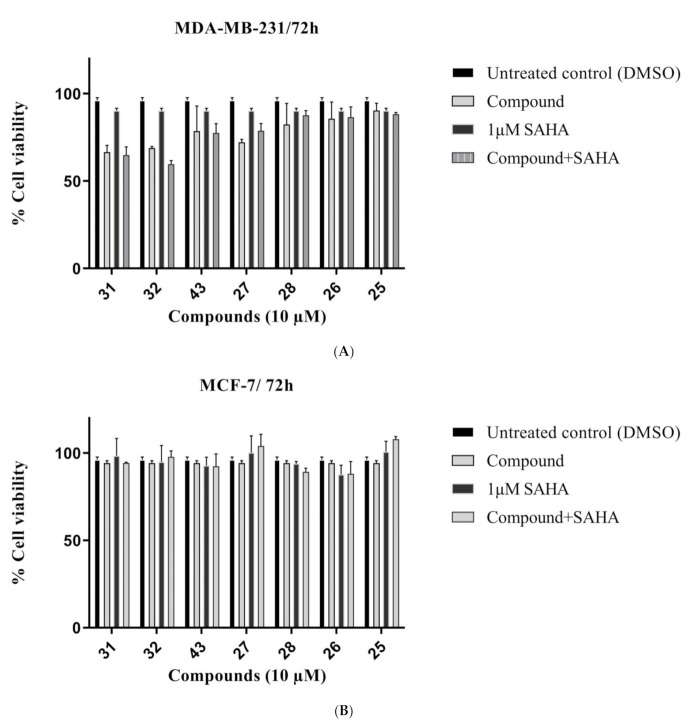
Cell viability of (**A**) MDA-MB-231 and (**B**) MCF-7 breast cancer cells treated with 10 μM compounds over 72 h, in the presence or absence of 1 μM of SAHA. Error bars represent mean values (±S.D.) for three independent determinations.

Against breast cancer cells, the furoindoles were more potent against the MDA-MB-231 cell line (Figure 5A). Furoindoles **31** and **32** displayed the highest activity against MDA-MB-231 cells with inhibition values of 34% and 31%, respectively. However, the compounds were less effective against MCF-7 cells, with viability being decreased by less than 10% for all compounds (Figure 5B). MCF-7 was determined as resistant cell line for SAHA alone treatment. The combination therapy also did not show enhancement activity.

#### 2.2.2. SAR Study of Furoindoles

Initial structure–activity relationship (SAR) analysis revealed that tetracyclic furoindoles (**31–32**) were more potent than the tricyclic furoindoles (**25**–**28**, **43**) against MDA-MB-231 and SH-SY5Y cells, although both classes showed similar cytotoxicity against MCF-7 and Kelly cells. Compound **31**, a tetracyclic furoindole bearing a single methyl substituent on both furo rings and the dimethyl substituted analogue **32** showed comparable reduction of cell viability against SH-SY5Y cells with the values of 35% and 30%, respectively (Figure 4B). Compounds **31** and **32** also showed good cytotoxicity against the MDA-MB-231 cell line with 35% and 32% reduction of cell viability, respectively (Figure 5A). This suggests that having a second furo ring fused to the indole moiety is beneficial for cytotoxic activity against MDA-MB-231 and SH-SY5Y cells.

The tricyclic furoindoles with a methoxy substituent on the indole benzene ring (**25**–**28**) showed more cytotoxic potency against neuroblastoma cancer cells than against the breast cancer cells. Furo[3,2-*e*]indoles **26** and **25** demonstrated similar levels of cytotoxicity against SH-SY5Y cells with inhibition values of 29% and 25%, respectively (Figure 4B), while against Kelly cells the inhibition values increased to 37% and 35% (Figure 4A). Furo[2,3-*g*]indoles **27** and **28** were found to be as efficient as furo[3,2-*e*]indoles. Compound **27** displayed the highest reduction value of 33% against SH-SY5Y cells and 29% against Kelly cells, while compound **28** inhibited SH-SY5Y and Kelly cells by 23% and 31%, respectively. Moreover, the two compounds showed different activity against MDA-MB-231 cells, with the monomethyl-substituted furoindole **27** (28% inhibition) being more potent than the dimethyl-substituted furoindole **28** (15% inhibition) (Figure 5A). This suggests that the number of methyl groups might modulate the cytototoxic activity of these compounds against certain cell lines. Interestingly, replacement of the methoxy group with a hydroxy substituent and including an additional trifluoromethyl group on the indole benzene ring (**43**) increased the activity of SAHA against the SH-SY5Y cells by 29% compared to SAHA alone (70%). The individual treatment of **43** provided 32% inhibition of the same cell line.

None of the tetracyclic or tricyclic compounds reduced MCF-7 cell viability by more than 10%, suggesting that those cells were the most resistant against the furoindole systems, the same as the SAHA (Figure 5B).

#### 2.2.3. Investigation of Toxicity Levels of Non-Cyclized Intermediate **30**, Tricyclic Furoindole **34** and Tetracylic Furoindole **32** against the Neuroblastoma Cells

**Figure 6:**
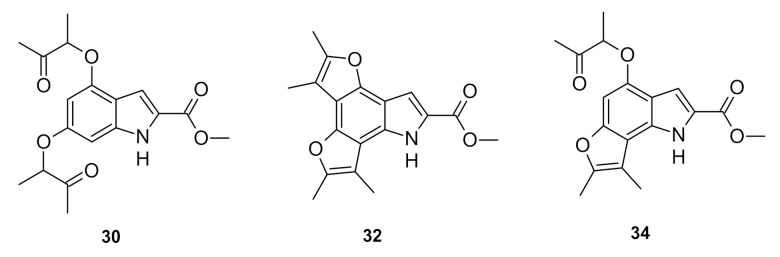
Compounds **30**, **32** and **34** against neuroblastoma cells.

Having confirmed the promising cytotoxic activity of tetracyclic furoindoles **31** and **32** against neuroblastoma cells, it was anticipated that the non-cyclized intermediate **30** and mono-cyclized tricyclic furoindole **34** (Figure 6) might also show cytotoxic efficiency against neuroblastoma cells.

**Figure 7:**
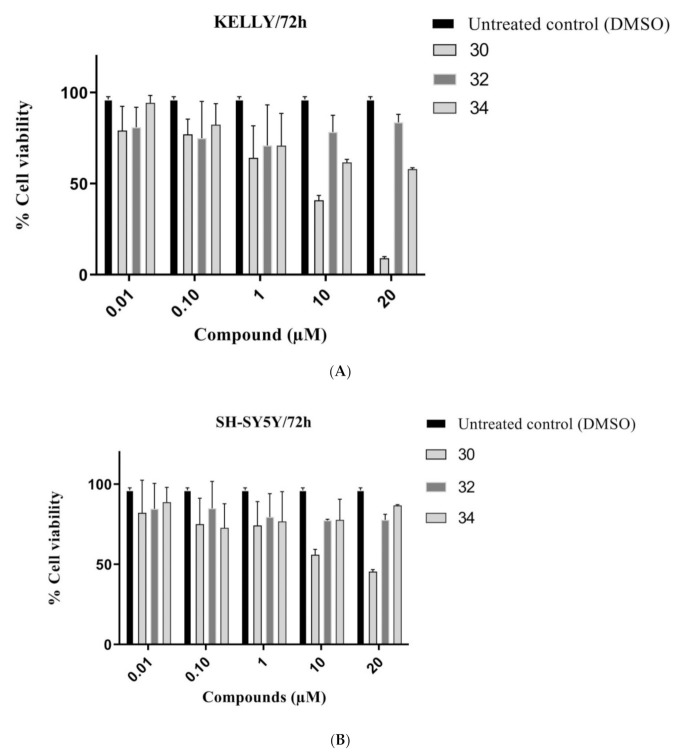
Cell viability of (**A**) KELLY (**B**) SH-SY5Y neuroblastoma cancer cells treated with compounds **30**, **32** and **34** at different concentrations (0.01, 0.1, 1, 10, 20 μM) over 72 h. Error bars represent mean values (±S.D.) for three independent determinations.

The IC_50_ values of furoindoles **30**, **32** and **34** against SH-SY5Y and Kelly cells were determined using the Alamar blue (Resazurin) assay, at concentrations of 0.01, 0.1, 1, 10, and 20 μM. The results showed that the non-cyclized compound **30** exhibited the greatest cytotoxic effects against neuroblastoma cells, with IC_50_ values of 13.3 μM and 3.1 μM towards SH-SY5Y and Kelly cells, respectively (Table 1) (Figure 7A,B). However, cyclization of the methyl ketone groups significantly decreased cytotoxic potency as the triyclic furoindole **34** and the tetracyclic furoindole **32** had IC_50_ values exceeding 20 μM for both neuroblastoma cell lines.

#### 2.2.4. Toxicity Study against Normal Cells

**Figure 8:**
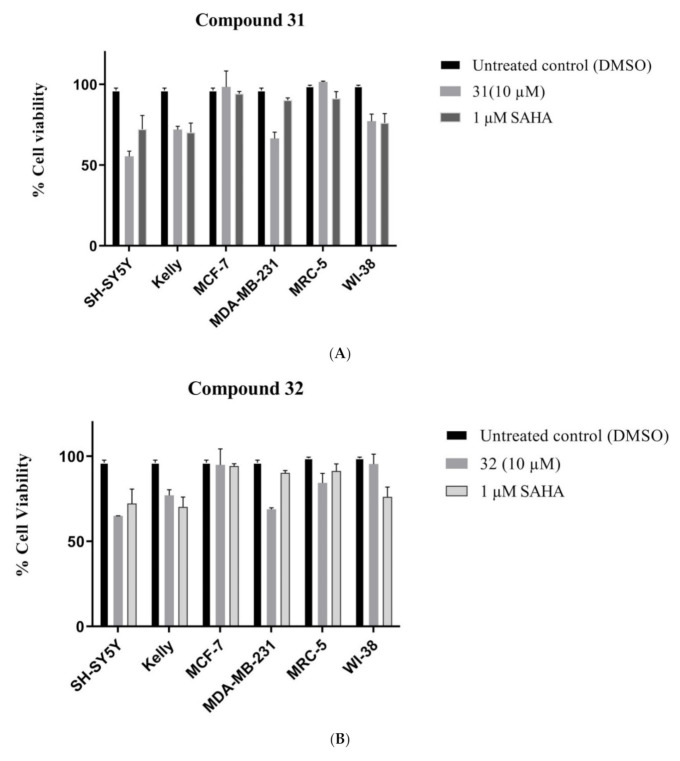

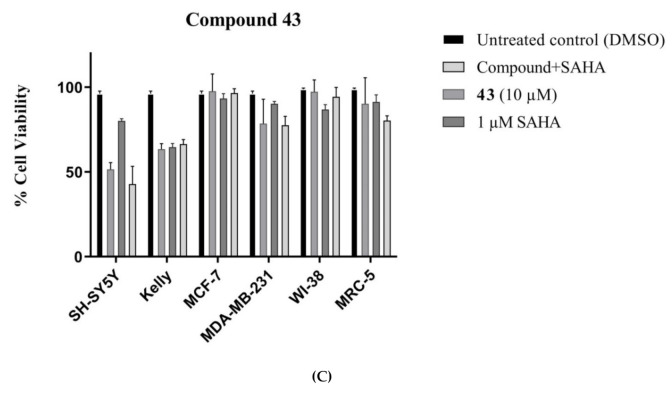
Comparative toxicity of compounds (**A**) **31**, **(B**) **32** and (**C**) **43** (10 µM) against cancer cells (SH-SY5Y, Kelly, MDA-MB-231 and MCF-7) and normal human fibroblasts (MRC-5 and WI-38) after 72 h exposure. Error bars represent mean values (±S.D.) for three independent determinations.

The most cytotoxic furoindoles, **31** and **32**, and the best candidate compound, **43**, for SAHA enhancement were screened against MRC-5 and WI-38 normal human lung fibroblast cells in order to investigate their level of selectivity for tumour cells. The toxicity of **31**, **32** and **43** towards the non-malignant cells were found to be slightly lower than towards the cancer cells. While compound **31** was the most toxic furoindole against normal cells with an inhibition value of 23% against WI-38 cells at 10 μM, it still showed higher cytotoxicity against SH-SY5Y cells with 45% inhibition at the same concentration (Figure 8A). The tetracyclic furoindole **32** reduced MRC-5 and WI-38 cell viability by 5% and 16%, respectively (Figure 8B). For compound **43**, it was toxic against the MRC-5 and WI-38 cell lines with 10% and 20% inhibitions; however, it was still more cytotoxic against Kelly cancer cells with at the same concentration of 10 μM (Figure 8C). The 1 μM SAHA treatment was also evaluated the normal human cells, and the SAHA was also found to be slightly toxic for MRC-5, but considerable inhibition was detected in the case of WI-38 cells.

**Figure 9:**
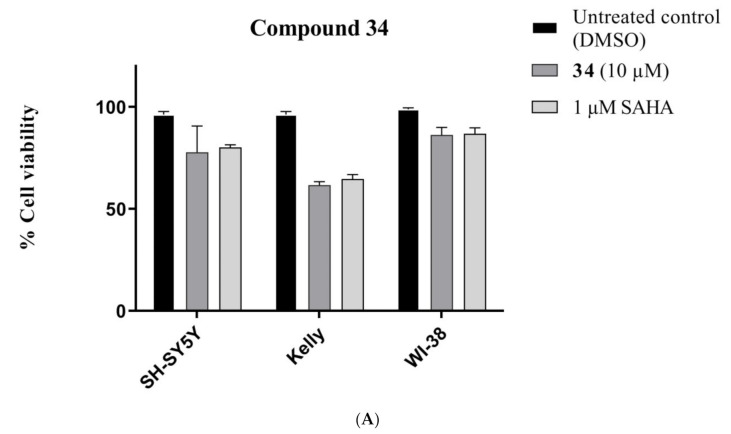

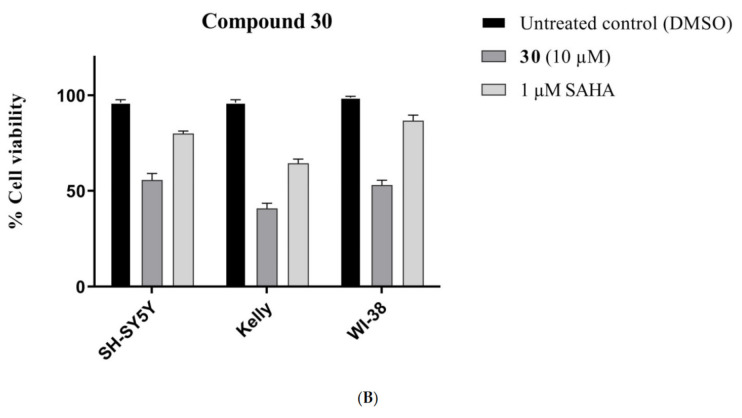
Comparative toxicity of (**A**) **34** and (**B**) **30** compounds (10 µM) against SH-SY5Y, Kelly and WI-38 cell lines after 72 h exposure. Error bars represent mean values (±S.D.) for three independent determinations.

Compounds **30** and **34** were also screened against WI-38 normal human cells to determine the effect of cyclization on the toxicity profile. Compound **34** showed a 15% reduction of WI-38 cell viability at a concentration of 10 μM (Figure 9A), while compound **30** caused 47% inhibition at the same concentration (Figure 9B). It was concluded that the cyclization process decreases the cytotoxic and toxic behaviours of the compounds against both the cancer and normal human cells.

## 3. Materials and Methods

### 3.1. General Information

Commercially available reagents were purchased from Fluka (Sydney, NSW, Australia), Aldrich (Sydney, NSW, Australia), Acros Organics (Morris Plains, NJ, USA), Alfa Aesar (Lancashire, UK) and Lancaster (Lancashire, UK) and purified if necessary. The synthetic procedures have been reported for all compounds as general methods and appropriate references have been given for known compounds. ^1^H (300 MHz) and ^13^C-NMR (75 MHz) spectra were obtained in the designated solvents on a DPX 300 spectrometer (Bruker, Sydney, NSW, Australia). Melting points were measured using a Mel-Temp melting point apparatus and are uncorrected. Infrared spectra were recorded on Avatar Series FT-IR spectrophotometer as KBr disks (Thermo Nicolet, Waltham, MA, USA). Ultraviolet spectra were measured using a Cary 100 spectrophotometer (Varian, Santa Clara, CA, USA) in the designated solvents and data reported as wavelength (λ) in nm and adsorption coefficient (ε) in cm^−1^M^−1^. High-resolution [ESI] mass spectra were recorded by the UNSW Bioanalytical Mass Spectrometry Facility, on an Orbitrap LTQ XL (Thermo Scientific, Waltham, MA, USA) ion trap mass spectrometer using a nanospray (nano-electrospray) ionization source.

*Methyl 4,6-dihydroxyindole-2-carboxylate* [22] (**17**): The *title* compound was prepared with the hydrogenolysis reaction from methyl 4,6-dibenzyloxyindole-2-carboxylate (**13**) (0.387 g, 1.0 mmol) and 5% Pd/C catalyst (40 mg) in methanol/THF mixture (15 mL) to give the product (149 mg, 72%) as a yellow solid; m.p. 246–248 °C (*lit*. [22] not given); ^1^H-NMR (300 MHz, *d*_6_-DMSO): δ 3.82 (s, 3H, CO_2_Me), 6.00 (dd, *J* = 2.3, 0.9 Hz, 1H, H5), 6.26 (dd, *J* = 2.3, 0.9 Hz, 1H, H7), 7.08 (d, *J* = 1.8 Hz, 1H, H3), 9.17 and 9.66 (bs, each 1H, OH), 11.31 (bs, 1H, NH).

*Methyl 5,6-dihydroxyindole-2-carboxylate* (**18**): The *title* compound was prepared with the hydrogenolysis reaction from methyl 5,6-dibenzyloxyindole-2-carboxylate (**14**) (0.387 g, 1.0 mmol) and 5% Pd/C catalyst (40 mg) in methanol/THF mixture (15 mL) to give the product (159 mg, 77%) as yellow solid; m.p. 256–258 °C; IR (KBr): *v*_max_ 3437, 3315, 2953, 2107, 1653, 1632, 1531, 1506, 1437, 1311, 1283, 1230, 1198, 1139, 992, 937, 849, 825, 767 cm^−1^; UV-vis (CH_3_CN): λ_max_ 203 nm (ε 42,100 cm^−1^ M^−1^), 318 (27,000); ^1^H-NMR (300 MHz, *d*_6_-DMSO): δ 3.82 (s, 3H, CO_2_Me), 6.79 (d, *J* = 0.8 Hz, 1H, H4), 6.88 (s, 1H, H3), 6.90 (d, *J* = 0.8 Hz, 1H, H7), 8.84 and 9.17 (bs, each 1H, OH), 11.28 (bs, 1H, NH); ^13^C-NMR (75.6 MHz, CDCl_3_): 52.0 (CO_2_Me), 97.3 (C7), 105.4 (C4), 108.4 (C3), 120.3 (ArC), 125.0 (C2), 133.2 (ArC), 142.3 (C5), 146.7 (C6), 162.5 (CO_2_Me); HRMS (+ESI): Found *m/z* 230.0423 [M + Na]^+^, C_10_H_9_NO_4_Na requires 230.0424.

*Methyl 5-hydroxy-6-methoxy-1H-indole-2-carboxylate* [23] (**19**): The *title* compound was prepared with the hydrogenolysis reaction from methyl 5-(benzyloxy)-6-methoxy-1*H*-indole-2-carboxylate (**15**) (0.311 g, 1.0 mmol) and 10% Pd/C catalyst (32 mg) in methanol/THF mixture (15 mL) to give the product (181 mg, 82%) as yellow solid; m.p. 176–178 °C (*lit*. [23] 178–180 °C); ^1^H-NMR (300 MHz, CDCl_3_): δ 3.95 (s, 3H, CO_2_Me), 3.99 (s, 3H, OMe), 5.57 (bs, 1H, OH), 6.86 (d, *J* = 0.9 Hz, 1H, H4), 7.11 (d, *J* = 2.1 Hz, 1H, H3), 7.16 (1H, s, H7), 8.77 (bs, 1H, NH).

*Methyl 6-hydroxy-5-methoxy-1H-indole-2-carboxylate* [24] (**20**): The *title* compound was prepared with the hydrogenolysis reaction from methyl 6-(benzyloxy)-5-methoxy-1*H*-indole-2-carboxylate (**16**) (0.311 g, 1.0 mmol) and 10% Pd/C catalyst (32 mg) in methanol/THF mixture (15 mL) to give the product (196 mg, 89%) as yellow solid; m.p. 184–186 °C (*lit*. [24] not given); ^1^H-NMR (300 MHz, CDCl_3_): δ 3.95 (s, 3H, CO_2_Me), 3.98 (s, 3H, OMe), 5.99 (bs, 1H, OH), 6.96 (d, *J* = 0.9 Hz, 1H, H7), 7.06 (s, 1H, H4), 7.14 (d, *J* = 2.1 Hz, 1H, H3), 8.86 (bs, 1H, NH).

#### 3.1.1. GP-1: General Procedure for the Synthesis of Indole Ethers

A mixture of methyl hydroxyindole-2-carboxylate (1 equiv.) and α-haloketone (per hydroxyl group, 1 equiv.) in acetone (100 mL) was treated with potassium carbonate (per hydroxyl group, 1 equiv.) and heated at reflux overnight. Acetone was removed under reduced pressure, and water (100 mL) was added to the residue. The product was extracted with ethyl acetate (3 × 100 mL), and the combined organic phases were dried over anhydrous sodium sulphate and concentrated under vacuum. The crude product was purified using gravity column chromatography, eluted with ethylacetate/*n*-hexane (1:10), to give the indole ethers.

#### 3.1.2. GP-2: General Procedure for the Synthesis of Furoindoles

A solution of indole ether (0.26 mmol) in DCM (10 mL) was treated with trifluoroacetic acid (10 drops) and heated at reflux for 24 h. The reaction mixture was poured into crushed ice (100 g), and the precipitate collected via filtration and washed with water. The crude product was purified using gravity column chromatography, eluted with DCM/*n*-hexane (2:10), to give the furoindole.

*Methyl 6-methoxy-5-(2-oxopropoxy)-1H-indole-2-carboxylate* (**21**): The *title* compound was prepared as described in GP-1 from methyl 5-hydroxy-6-methoxyindole-2-carboxylate (**19**) (107.2 mg, 0.485 mmol), chloroacetone (44.2 mg, 0.485 mmol) and potassium carbonate (66.9 mg, 0.485 mmol) to give the product (108.8 mg, 81%) as a white solid; m.p. 164–166 °C; IR (KBr): *ν*_max_ 3314, 2879, 2828, 2318, 2117, 1925, 1688, 1632, 1521, 1445, 1356, 1250, 1151, 1065, 1019, 998, 893, 759, 671 cm^−1^; UV-vis (CH_3_CN): λ_max_ 208 nm (ε 30,600 cm^−1^ M^−1^), 318 (22,100); ^1^H-NMR (300 MHz, CDCl_3_): δ 2.34 (s, 3H, COMe), 3.95 (s, 3H, CO_2_Me), 3.97 (s, 3H, OMe), 4.64 (s, 2H, CH_2_), 6.91 (d, *J* = 0.9 Hz, 1H, H7), 7.02 (s, 1H, H3), 7.11 (d, *J* = 0.9 Hz, 1H, H4), 8.90 (bs, 1H, NH); ^13^C-NMR (75.6 MHz, CDCl_3_): δ 26.5 (COMe), 51.9 (CO_2_Me), 56.1 (Ome), 75.0 (CH_2_), 94.4 (C7), 106.0 (C4), 108.9 (C3), 120.3 (aryl C), 126.9 (C2), 132.9 (aryl C), 144.5 (C5), 150.4 (C6), 162.2 (CO_2_Me), 206.5 (COMe); HRMS (+ESI): Found *m/z* 300.0832, [M + Na]^+^, C_14_H_15_NO_5_Na requires 300.0842.

*Methyl 5-methoxy-6-(2-oxopropoxy)-1H-indole-2-carboxylate* (**23**): The *title* compound was prepared as described in **GP-1** from methyl 5-methoxy-6-hydroxyindole-2-carboxylate (**20**) (107.2 mg, 0.485 mmol), chloroacetone (44.2 mg, 0.485 mmol) and potassium carbonate (66.9 mg, 0.485 mmol) to give the product (104.8 mg, 78%) as a white solid; m.p. 170–172 °C; IR (KBr): *ν*_max_ 3316, 2918, 2845, 2339, 2109, 1910, 1680, 1642, 1520, 1428, 1356, 1285, 1209, 1160, 1017, 890, 817, 761, 666 cm^−1^; UV-vis (CH_3_CN): λ_max_ 209 nm (ε 30,700 cm^−1^ M^−1^), 309 (18,500); ^1^H-NMR (300 MHz, CDCl_3_): δ 2.33 (s, 3H, COMe), 3.94 (s, 3H, CO_2_Me), 3.95 (s, 3H, OMe), 4.65 (s, 2H, CH_2_), 6.80 (d, *J* = 0.7 Hz, 1H, H4), 7.12 (s, 1H, H3), 7.13 (d, *J* = 0.7 Hz, 1H, H7), 9.05 (bs, 1H, NH); ^13^C-NMR (75.6 MHz, CDCl_3_): δ26.5 (COMe), 51.8 (CO_2_Me), 56.2 (OMe), 74.5 (CH_2_), 95.5 (C7), 103.4 (C4), 108.6 (C3), 121.6 (aryl C), 126.3 (C2), 131.6 (aryl C), 146.4 (C6), 148.1 (C5), 162.2 (CO_2_Me), 206.3 (COMe); HRMS (+ESI): Found *m/z* 300.0842, [M + Na]^+^, C_14_H_15_NO_5_Na requires 300.0842.

*Methyl 6-methoxy-5-((3-oxobutan-2-yl)oxy)-1H-indole-2-carboxylate* (**22**): The *title* compound was prepared as described in **GP-1** from methyl 5-hydroxy-6-methoxyindole-2-carboxylate (**19**) (107.2 mg, 0.485 mmol), 3-chloro-2-butanone (51.4 mg, 0.485 mmol), potassium carbonate (66.9 mg, 0.485 mmol) to give the product (120 mg, 85%) as a white solid; m.p. 134–136 °C; IR (KBr): *ν*_max_ 3308, 2928, 2802, 2287, 2116, 1965, 1676, 1622, 1519, 1440, 1358, 1274, 1191, 1142, 1092, 1013, 937, 820, 766, 688 cm^−1^; UV-vis (CH_3_CN): λ_max_ 207 nm (ε 31,400 cm^−1^ M^−1^), 319 (21,700); ^1^H-NMR (300 MHz, CDCl_3_): δ 1.56 (d, *J* = 6.9 Hz, 3H, CHMe), 2.29 (s, 3H, COMe), 3.94 (s, 3H, CO_2_Me), 3.95 (s, 3H, OMe), 4.57 (q, *J* = 6.9 Hz, 1H, CHMe), 6.90 (d, *J* = 0.8 Hz, 1H, H7), 7.04 (s, 1H, H3), 7.10 (d, *J* = 0.9 Hz, 1H, H4), 8.92 (bs, 1H, NH); ^13^C-NMR (75.6 MHz, CDCl_3_): δ 17.6 (CHMe), 24.7 (COMe), 51.8 (CO_2_Me), 56.0 (Ome), 81.4 (CHMe), 94.4 (C7), 107.8 (C4), 108.9 (C3), 120.3 (aryl C), 126.1 (C2), 133.0 (aryl C), 143.9 (C6), 150.8 (C5), 162.2 (CO_2_Me), 211.0 (COMe); HRMS (+ESI): Found *m/z* 314.0988, [M + Na]^+^, C_15_H_17_NO_5_Na requires 314.0999.

*Methyl 5-methoxy-6-((3-oxobutan-2-yl)oxy)-1H-indole-2-carboxylate* (**24**): The *title* compound was prepared as described in **GP-1** from methyl 5-methoxy-6-hydroxyindole-2-carboxylate (**20**) (107.2 mg, 0.485 mmol), 3-chloro-2-butanone (51.4 mg, 0.485 mmol), potassium carbonate (66.9 mg, 0.485 mmol) to give the product (114 mg, 81%) as a white solid; m.p. 136–138 °C; IR (KBr): *ν*_max_ 3321, 2915, 2842, 2327, 2108, 1928, 1678, 1640, 1520, 1438, 1354, 1288, 1221, 1101, 1022, 937, 848, 830, 766, 672 cm^−1^; UV-vis (CH_3_CN): λ_max_ 210 nm (ε 34,200 cm^−1^ M^−1^), 289 (20,300); ^1^H-NMR (300 MHz, CDCl_3_): δ 1.58 (d, *J* = 6.9 Hz, 3H, CHMe), 2.26 (s, 3H, COMe), 3.92 (s, 3H, CO_2_Me), 3.95 (s, 3H, OMe) 4.63 (q, *J* = 6.9 Hz, 1H, CHMe), 6.80 (d, *J* = 0.9 Hz, 1H, H4), 7.12 (s, 1H, H3), 7.14 (d, *J* = 0.9 Hz, 1H, H7), 8.87 (bs, 1H, NH); ^13^C-NMR (75.6 MHz, CDCl_3_):δ 17.6 (CHMe), 24.5 (COMe), 51.8 (CO_2_Me), 56.2 (OMe), 81.0 (CHMe), 97.8 (C7), 103.6 (C4), 108.6 (C3), 121.7 (aryl C), 126.4 (C2), 131.6 (aryl C), 146.7 (C6), 147.8 (C5), 162.1 (CO_2_Me), 210.8 (COMe); HRMS (+ESI): Found *m/z* 314.0991, [M + Na]^+^, C_15_H_17_NO_5_Na requires 314.0999.

*Methyl 4-methoxy-1-methyl-6H-furo[3,2-e]indole-7-carboxylate* (**25**): The *title* compound was prepared as described in **GP-2** from methyl 6-methoxy-5-(2-oxopropoxy)-1*H*-indole-2-carboxylate (**21**) (72.0 mg, 0.26 mmol) with trifluoroacetic acid (10 drops) in dichloromethane (10 mL) to give the product (49.1 mg, 73%) as a white solid; m.p. 202–204 °C; IR (KBr): *ν*_max_ 3289, 2919, 2857, 2342, 2113, 1933, 1673, 1621, 1521, 1439, 1391, 1280, 1186, 1144, 1083, 1030, 997, 916, 761, 693 cm^−1^; UV-vis (CH_3_CN): λ_max_ 209 nm (ε 33,800 cm^−1^ M^−1^), 312 (37.900), 324 (37,500); ^1^H-NMR (300 MHz, CDCl_3_): δ 2.48 (d, *J* = 1.1 Hz, 3H, Me), 3.97 (s, 3H, CO_2_Me), 4.05 (s, 3H, OMe), 6.77 (s, 1H, H5), 7.46 (d, *J* = 1.4 Hz, 1H, H8), 7.51 (d, *J* = 1.1 Hz, 1H, H2), 9.03 (bs, 1H, NH); ^13^C-NMR (75.6 MHz, CDCl_3_): δ 9.3 (C-Me), 51.8 (CO_2_Me), 56.1 (Ome), 107.1 (C8), 114.4 (aryl C), 116.9 (C5), 122.5 (C1), 125.1 (aryl C), 134.3 (C7), 140.8 (aryl C), 141.4 (aryl C), 142.9 (C2), 146.1 (C4), 162.3 (CO_2_Me); HRMS (+ESI): Found *m/z* 282.0731, [M + Na]^+^, C_14_H_13_NO_4_Na requires 282.0737.

*Methyl 5-methoxy-8-methyl-1H-furo[2,3-g]indole-2-carboxylate* (**27**): The *title* compound was prepared as described in **GP-2** from methyl 5-methoxy-6-(2-oxopropoxy)-1*H*-indole-2-carboxylate (**23**) (72.0 mg, 0.26 mmol) with trifluoroacetic acid (10 drops) in dichloromethane (10 mL) to give the product (41.1 mg, 61%) as a white solid; m.p. 182–184 °C; IR (KBr): *ν*_max_ 3294, 2919, 2849, 2370, 2119, 1917, 1683, 1625, 1532, 1433, 1380, 1290, 1196, 1146, 1041, 989, 920, 823, 746, 674 cm^−1^; UV-vis (CH_3_CN): λ_max_ 213 nm (ε 20,200 cm^−1^ M^−1^), 250 (21,900), 294 (16,500); ^1^H-NMR (300 MHz, CDCl_3_): δ 2.53 (d, *J* = 1.3 Hz, 3H, Me), 3.98 (s, 3H, CO_2_Me), 4.05 (s, 3H, OMe), 6.97 (s, 1H, H4), 7.28 (d, *J* = 3.0 Hz, 1H, H3), 7.49 (d, *J* = 1.3 Hz, 1H, H7), 9.12 (bs, 1H, NH), ^13^C-NMR (75.6 MHz, CDCl_3_): δ 9.7 (C-Me), 51.8 (CO_2_Me), 56.1 (OMe), 109.7 (C3), 114.8 (C4), 115.1 (C8), 122.8 (C2), 125.4 (aryl C), 125.7 (aryl C), 140.8 (2×aryl C), 142.9 (C7), 145.5 (C5), 162.3 (CO_2_Me); HRMS (+ESI): Found *m/z* 282.0730, [M + Na]^+^, C_14_H_13_NO_4_Na requires 282.0737.

*Methyl 4-methoxy-1,2-dimethyl-6H-furo[3,2-e]indole-7-carboxylate* (**26**): The *title* compound was prepared as described in **GP-2** from methyl 6-methoxy-5-((3-oxobutan-2-yl)oxy)-1*H*-indole-2-carboxylate (**22**) (75.7 mg, 0.26 mmol) with the catalytic amount of trifluoroacetic acid (10 drops) in dichloromethane (10 mL) to give the product (56.1 mg, 79%) as a white solid; m.p. 182–184 °C; IR (KBr): *ν*_max_ 3308, 2939, 2855, 2342, 2116, 1933, 1671, 1625, 1520, 1438, 1371, 1280, 1211, 1133, 1035, 1012, 927, 807, 684 cm^−1^; UV-vis (CH_3_CN): λ_max_ 211 nm (ε 31,400 cm^−1^ M^−1^), 314 (38,400); ^1^H-NMR (300 MHz, CDCl_3_): δ 2.41 (d, *J* = 0.8 Hz, 3H, Me), 2.48 (d, *J* = 0.8 Hz, 3H, Me), 3.98 (s, 3H, CO_2_Me), 4.06 (s, 3H, OMe), 6.72 (s, 1H, H5), 7.46 (d, *J* = 0.9 Hz, 1H, H8), 8.96 (bs, 1H, NH); ^13^C-NMR (75.6 MHz, CDCl_3_): δ 9.4 (Me), 11.8 (Me), 51.7 (CO_2_Me), 56.0 (Ome), 107.1 (C1and aryl C), 111.2 (C8), 114.2 (C5), 123.7 (aryl C), 124.9 (C7), 134.4 (aryl C), 139.6 (C4), 145.7 (aryl C), 150.6 (C2), 162.3 (CO_2_Me); HRMS (+ESI): Found *m/z* 296.0889, [M + Na]^+^, C_15_H_15_NO_4_Na requires 296.0893.

*Methyl 5-methoxy-7,8-dimethyl-1H-furo[2,3-g]indole-2-carboxylate* (**28**): The *title* compound was prepared as described in **GP-2** from methyl 5-methoxy-6-((3-oxobutan-2-yl)oxy)-1*H*-indole-2-carboxylate (**24**) (75.7 mg, 0.26 mmol) with the catalytic amount of trifluoroacetic acid (10 drops) in dichloromethane (10 mL) to give the product (47.6 mg, 67%) as a white solid; m.p. 176–178 °C; IR (KBr): *ν*_max_ 3297, 2917, 2845, 2342, 2092, 1942, 1684, 1593, 1529, 1431, 1364, 1293, 1236, 1192, 1048, 995, 928, 829, 766, 670 cm^−1^; UV-vis (CH_3_CN): λ_max_ 215 nm (ε 29,500 cm^−1^ M^−1^), 251 (33,800), 299 (22.300); ^1^H-NMR (300 MHz, CDCl_3_): δ 2.45 (d, *J* = 0.8 Hz, 3H, Me), 2.47 (d, *J* = 0.8 Hz, 3H, Me), 3.97 (s, 3H, CO_2_Me), 4.05 (s, 3H, OMe), 6.90 (s, 1H, H4), 7.26 (d, *J* = 2.2 Hz, 1H, H3), 9.06 (bs, 1H, NH); ^13^C-NMR (75.6 MHz, CDCl_3_): δ 9.4 (Me), 11.8 (Me), 51.7 (CO_2_Me), 56.0 (OMe), 107.1 (C3), 111.2 (C4), 114.2 (C8), 123.7 (aryl C), 124.9 (aryl C), 127.0 (C2), 129.1 (aryl C), 134.4 (aryl C), 145.7 (C5), 150.6 (C7), 162.3 (CO_2_Me); HRMS (+ESI): Found *m/z* 274.1071, [M + H]^+^, C_15_H_16_NO_4_ requires 274.1074.

*Methyl 4,6-bis(2-oxopropoxy)-1H-indole-2-carboxylate* (**29**): The *title* compound was prepared as described in **GP-1** from methyl 4,6-dihdroxyindole-2-carboxylate (**17**) (100.4 mg, 0.485 mmol), chloroacetone (88.4 mg, 0.97 mmol), potassium carbonate (123.8 mg, 0.97 mmol) to give the product (126.9 mg, 82%) as a white solid; m.p. 184–186 °C; IR (KBr): *ν*_max_ 3305, 2919, 2822, 2341, 2107, 1898, 1688, 1626, 1584, 1520, 1435, 1354, 1275, 1200, 1144, 985, 951, 803, 767 cm^−1^; UV-vis (CH_3_CN): λ_max_ 203 nm (ε 30,400 cm^−1^ M^−1^), 245 (23,000), 305 (23,200); ^1^H-NMR (300 MHz, CDCl_3_): δ 2.33 (s, 3H, Me), 2.39 (s, 3H, Me), 3.95 (s, 3H, CO_2_Me), 4.59 (d, *J* = 0.8 Hz, 2H, CH_2_), 4.67 (d, *J* = 0.8 Hz, 2H, CH_2_), 6.28 (d, *J* = 1.7 Hz, 1H, H5), 6.37 (d, *J* = 1.7 Hz, 1H, H7), 7.26 (d, *J* = 2.2 Hz, 1H, H3), 8.96 (bs, 1H, NH); ^13^C-NMR (75.6 MHz, CDCl_3_): δ30.9 (2 × COMe), 51.9 (CO_2_Me), 73.0 (O-CH_2_), 73.4 (O-CH_2_), 88.1 (C5), 93.8 (C7), 106.6 (C3), 114.3 (aryl C), 125.4 (C2), 138.0 (aryl C), 153.2 (C4), 157.9 (C6), 162.0 (CO_2_Me), 205.5 (COMe), 207.0 (COMe); HRMS (+ESI): Found *m/z* 342.0948, [M + Na]^+^, C_16_H_17_NO_6_Na requires 342.0936.

*Methyl 4,6-bis((3-oxobutan-2-yl)oxy)-1H-indole-2-carboxylate* (**30**): The *title* compound was prepared as described in **GP-1** from methyl 4,6-dihdroxyindole-2-carboxylate (**17**) (100.4 mg, 0.485 mmol), 3-chloro-2-butanone (102.8 mg, 0.97 mmol), potassium carbonate (123.8 mg, 0.97 mmol) to give the product (148.1 mg, 88%) as a white solid; m.p. 166–168 °C; IR (KBr): *ν*_max_ 3309, 2918, 2846, 2315, 2112, 1917, 1683, 1621, 1584, 1520, 1438, 1380, 1280, 1205, 1160, 1088, 991, 934, 811, 769, 685 cm^−1^; UV-vis (CH_3_CN): λ_max_ 207 nm (ε 28,500 cm^−1^ M^−1^), 245 (21,600), 305 (22,800); ^1^H-NMR (300 MHz, CDCl_3_): δ 1.52 (d, *J* = 6.9 Hz, 3H, CH-Me), 1.59 (d, *J* = 6.9 Hz, 3H, CH-Me), 2.16 (s, 3H, COMe), 2.24 (s, 3H, COMe), 3.95 (s, 3H, CO_2_Me), 4.55 (q, *J* = 6.9 Hz, 1H, CH-Me), 4.78 (q, *J* = 6.9 Hz, 1H, CH-Me), 6.09 (d, *J* = 1.8 Hz, 1H, H5) 6.32 (d, *J* = 1.8 Hz, 1H, H7), 7.32 (t, *J* = 1.1 Hz, 1H, H3), 8.85 (bs, 1H, NH); ^13^C-NMR (75.6 MHz, CDCl_3_): δ17.4 (CH-Me), 17.6 (CH-Me), 24.4 (COMe), 24.6 (COMe), 51.6 (CO_2_Me), 79.9 (C5), 88.5 (C7), 94.5 (CH-Me), 94.6 (CH-Me), 109.2 (C3), 112.9 (aryl C), 125.3 (C2), 141.1 (aryl C), 153.2 (C4), 158.0 (C6), 162.3 (CO_2_Me), 202.6 (COMe), 210.6 (COMe); HRMS (+ESI): Found *m/z* 370.1260, [M + Na]^+^, C_18_H_21_NO_6_Na requires 370.1267.

*Methyl 8-methyl-4-(2-oxopropoxy)-1H-furo[2,3-g]indole-2-carboxylate* (**33**): The *title* compound was prepared as described in **GP-2** from methyl 4,6-bis(2-oxopropoxy)-1*H*-indole-2-carboxylate (**29**) (82.9 mg, 0.26 mmol) with trifluoroacetic acid (10 drops) in dichloromethane (10 mL) to give the product (11.7 mg, 15%) as a white solid; m.p. 142–144 °C; IR (KBr): *ν*_max_ 3357, 2916, 2848, 2340, 2120, 1927, 1700, 1635, 1560, 1506, 1435, 1355, 1280, 1149, 1093, 1050, 994, 940, 801, 770, 704 cm^−1^; UV-vis (CH_3_CN): λ_max_ 208 nm (ε 16,600 cm^−1^ M^−1^), 261 (40,200), 298 (15,100); ^1^H-NMR (300 MHz, CDCl_3_): δ 2.40, (s, 3H, COMe), 2.52 (d, *J* = 1.3 Hz, 3H, C-Me), 4.00 (s, 3H, CO_2_Me), 4.70 (s, 2H, CH_2_), 6.63 (s, 1H, H5), 7.39 (q, *J* = 1.3 Hz, 1H, H7), 7.52 (d, *J* = 2.2 Hz, 1H, H3), 9.14 (bs, 1H, NH); ^13^C-NMR (75.6 MHz, CDCl_3_):δ 9.8 (C-Me), 26.7 (COMe), 51.9 (CO_2_Me), 73.3 (CH_2_), 88.7 (C5), 107.7 (C3), 108.4 (aryl C), 114.3 (C8), 115.5 (aryl C), 124.9 (C2), 130.8 (aryl C), 139.6 (C7), 150.3 (C4), 155.3 (aryl C), 162.2 (CO_2_Me), 207.9 (COMe); HRMS (+ESI): Found *m/z* 324.0846, [M + Na]^+^, C_16_H_15_NO_5_Na requires 324.0848.

*Methyl 3,6-dimethyl-7H-difuro[2,3-e:2′,3′-g]indole-8-carboxylate* (**31**): The *title* compound was prepared as described in **GP-2** from methyl 4,6-bis(2-oxopropoxy)-1*H*-indole-2-carboxylate (**29**) (82.9 mg, 0.26 mmol) with trifluoroacetic acid (10 drops) in dichloromethane (10 mL) to give the product (38.3 mg, 52%) as a white solid; m.p. 182–184 °C; IR (KBr): *ν*_max_ 3292, 2918, 2849, 2318, 2116, 1917, 1676, 1641, 1524, 1432, 1400, 1343, 1281, 1298, 1103, 990, 924, 875, 800, 688 cm^−1^; UV-vis (CH_3_CN): λ_max_ 238 nm (ε 33,400 cm^−1^ M^−1^), 255 (31,200), 299 (15,400); ^1^H-NMR (300 MHz, CDCl_3_): δ 2.55 (d, *J* = 1.3 Hz, 3H, Me), 2.57 (d, *J* = 1.3 Hz, 3H, Me), 3.99 (s, 3H, CO_2_Me), 7.43 (d, *J* = 1.3 Hz, 1H, H5), 7.48 (d, *J* = 1.3 Hz, 1H, H2), 7.55 (d, *J* = 2.1 Hz, 1H, H9), 9.20 (bs, 1H, NH), ^13^C-NMR (75.6 MHz, CDCl_3_): 9.4 (CH-Me), 9.9 (CH-Me), 51.9 (CO_2_Me), 105.8 (C3 and C6), 109.5 (C9), 110.0 (aryl C), 111.1 (aryl C), 114.3 (aryl C), 115.2 (aryl C), 124.9 (C8), 129.0 (aryl C), 139.1 (C5), 139.4 (C2), 147.8 (aryl C), 162.2 (CO_2_Me); HRMS (+ESI): Found *m/z* 306.0733, [M + Na]^+^, C_16_H_13_NO_4_Na requires 306.0737.

*Methyl 7,8-dimethyl-4-((3-oxobutan-2-yl)oxy)-1H-furo[2,3-g]indole-2-carboxylate* (**34**): The *title* compound was prepared as described in **GP-2** from methyl 4,6-bis((3-oxobutan-2-yl)oxy)-1H-indole-2-carboxylate (**30**) (90.2 mg, 0.26 mmol) with the catalytic amount of trifluoroacetic acid (10 drops) in dichloromethane (10 mL) to give the product (18 mg, 21%) as a white solid; m.p. 150–152 °C; IR (KBr): *ν*_max_ 3326, 2921, 2831, 2338, 2110, 1907, 1686, 1628, 1590, 1528, 1501, 1437, 1354, 1271, 1161, 1115, 994, 941, 795, 770, 747 cm^−1^; UV-vis (CH_3_CN): λ_max_ 263 nm (ε 58,400 cm^−1^ M^−1^), 301 (21,000); ^1^H-NMR (300 MHz, CDCl_3_): δ 1.62 (d, *J* = 7.0 Hz, 3H, CH-Me), 1.64 (s, 3H, COMe), 2.40 (s, 3H, C-Me), 2.42 (s, 3H, C-Me), 3.99 (s, 3H, CO_2_Me), 4.77 (q, *J* = 7.0 Hz, 1H, CH-Me), 6.65 (s, 1H, H5), 7.49 (d, *J* = 2.2 Hz, 1H, H3), 9.02 (bs, 1H, NH); ^13^C-NMR (75.6 MHz, CDCl_3_): δ9.9 (C-Me), 11.5 (C-Me), 17.6 (CH-Me), 29.7 (CO-Me), 51.9 (CO_2_Me), 79.5 (CH-Me), 89.1 (C5), 107.8 (C3), 108.2 (aryl C), 115.7 (C8), 122.7 (aryl C), 124.6 (C2), 131.3 (aryl C), 148.4 (C4), 149.0 (C7), 153.2 (aryl C), 162.8 (CO_2_Me), 210.6 (CO-Me); HRMS (+ESI): Found *m/z* 352.1154, [M + Na]^+^, C_18_H_19_NO_5_Na requires 352.1161.

*Methyl 2,3,5,6-tetramethyl-7H-difuro[2,3-e:2′,3′-g]indole-8-carboxylate* (**32**): The *title* compound was prepared as described in **GP-2** from methyl 4,6-bis((3-oxobutan-2-yl)oxy)-1*H*-indole-2-carboxylate (**30**) (90.2 mg, 0.26 mmol) with trifluoroacetic acid (10 drops) in dichloromethane (10 mL) to give the product (49.3 mg, 61%) as a white solid; m.p. 226–228 °C; IR (KBr): *ν*_max_ 3329, 2918, 2864, 2318, 2105, 1921, 1680, 1639, 1528, 1437, 1368, 1275, 1298, 1180, 1080, 998, 945, 800, 749, 651 cm^−1^; UV-vis (CH_3_CN): λ_max_ 238 nm (ε 33,600 cm^−1^ M^−1^), 259 (30,600), 304 (14,200); ^1^H-NMR (300 MHz, CDCl_3_): δ 2.44 (s, 3H, Me), 2.45 (s, 3H, Me), 2.46 (s, 3H, Me), 2.47 (s, 3H, Me), 3.97 (s, 3H, CO_2_Me), 7.49 (d, *J* = 2.1 Hz, 1H, H9), 9.08 (bs, 1H, NH); ^13^C-NMR (75.6 MHz, CDCl_3_): δ9.5 (C-Me), 10.0 (C-Me), 11.6 (2×C-Me), 51.8 (CO_2_Me), 105.4 (C3 and C6), 108.6 (C9), 108.9 (aryl C), 110.2 (aryl C), 110.7 (aryl C), 110.7 (aryl C), 124.6 (C8), 128.3 (aryl C), 146.5 (aryl C),147.7 (C2), 147.9 (C5), 162.4 (CO_2_Me); HRMS (+ESI): Found *m/z* 312.1232, [M + H]^+^, C_18_H_18_NO_4_ requires 312.1230.

*Methyl 5,6-bis(2-oxopropoxy)-1H-indole-2-carboxylate* (**37**): The title compound was prepared as described in **GP-1** from methyl 5,6-dihdroxyindole-2-carboxylate (**18**) (100.4 mg, 0.485 mmol), chloroacetone (88.4 mg, 0.97 mmol), potassium carbonate (123.8 mg, 0.97 mmol) to give the product (100.6 mg, 65%) as a white solid; m.p. 138–140 °C; IR (KBr): *ν*_max_ 3326, 2915, 2849, 2362, 2137, 1966, 1677, 1633, 1522, 1480, 1434, 1357, 1244, 1207, 1145, 1041, 991, 897, 842, 761, 676 cm^−1^; UV-vis (CH_3_CN): λ_max_ 209 nm (ε 35,500 cm^−1^ M^−1^), 310 (23,900); ^1^H-NMR (300 MHz, CDCl_3_): δ 2.36 (s, 6H, 2 × Me), 3.95 (s, 3H, CO_2_Me), 4.59 (s, 2H, CH_2_), 4.67 (s, 2H, CH_2_), 6.84 (d, *J* = 0.6 Hz, 1H, H4), 7.08 (s, 1H, H3), 7.12 (dd, *J* = 2.1, 0.9 Hz, 1H, H7), 8.89 (bs, 1H, NH), ^13^C-NMR (75.6 MHz, CDCl_3_): δ 26.6 (CO-Me), 29.7 (CO-Me), 51.9 (CO_2_Me), 74.3 (CH_2_), 74.8 (CH_2_), 98.3 (C7), 106.5 (C4), 108.5 (C3), 121.5 (aryl C), 126.8 (C2), 132.3 (aryl C), 144.5 (C6), 148.3 (C5), 162.0 (CO_2_Me), 205.5 (CO-Me), 205.8 (CO-Me); HRMS (+ESI): Found *m/z* 342.0940, [M + Na]^+^, C_16_H_17_NO_6_Na requires 342.0948.

*Methyl 5,6-bis((3-oxobutan-2-yl)oxy)-1H-indole-2-carboxylate* (**38**): The *title* compound was prepared as described in **GP-1** from methyl 5,6-dihydroxyindole-2-carboxylate (**18**) (100.4 mg, 0.485 mmol), 3-chloro-2-butanone (102.8 mg, 0.97 mmol), potassium carbonate (123.8 mg, 0.97 mmol) to give the product (114.5 mg, 68%) as a white solid; m.p. 120–122 °C; IR (KBr): *ν*_max_ 3335, 2917, 2832, 2359, 2100, 1895, 1687, 1597, 1519, 1468, 1434, 1350, 1252, 1208, 1162, 1082, 977, 919, 838, 755 cm^−1^; UV-vis (CH_3_CN): λ_max_ 212 nm (ε 34,100 cm^−1^ M^−1^), 305 (22,400); ^1^H-NMR (300 MHz, CDCl_3_): δ 1.51–1.57 (m, 6H, 2×CH-Me), 2.27 (d, *J* = 1.8 Hz, 3H, CO-Me), 2.31 (d, *J* = 1.8 Hz, 3H, CO-Me), 3.88 (s, 3H, CO_2_Me), 4.60–4.69 (m, 2H, 2×CH-Me), 6.59 (s, 1H, H3), 7.09 (q, *J* = 1.8 Hz, 1H, H7), 7.24 (q, *J* = 1.8 Hz, 1H, H4), 8.81 (bs, 1H, NH), ^13^C-NMR (75.6 MHz, CDCl_3_): δ 17.5 (CH-Me), 17.6 (CH-Me), 24.6 (CO-Me), 24.8 (CO-Me), 51.6 (CO_2_Me), 81.4 (C7), 96.5 (C4), 108.7 (CH-Me), 108.9 (CH-Me), 111.0 (C3), 120.2 (aryl C), 126.6 (C2), 135.2 (aryl C), 144.6 (C6), 148.5 (C5), 162.3 (CO_2_Me), 202.7 (2 × CO-Me); HRMS (+ESI): Found *m/z* 370.1254, [M + Na]^+^, C_18_H_21_NO_6_Na requires 370.1261.

*Methyl 5-hydroxy-6-(2-oxopropoxy)-1H-indole-2-carboxylate* (**41**): The title compound was prepared as described in **GP-1** from methyl 5,6-dihydroxyindole-2-carboxylate (**18**) (100.4 mg, 0.485 mmol), chloroacetone (88.4 mg, 0.97 mmol), potassium carbonate (123.8 mg, 0.97 mmol) to give the product (35.7 mg, 28%) as a white solid; m.p. 182–184 °C; IR (KBr): *ν*_max_ 3334, 2918, 2855, 2318, 2106, 1948, 1682, 1622, 1519, 1486, 1430, 1351, 1248, 1203, 995, 920, 826, 799, 749, 694 cm^−1^; UV-vis (CH_3_CN): λ_max_ 238 nm (ε 37,500 cm^−1^ M^−1^), 259 (23,200);^1^H-NMR (300 MHz, CDCl_3_): δ 2.37 (s, 3H, Me), 3.90 (s, 3H, CO_2_Me), 4.67 (s, 2H, CH_2_), 5.26 (bs, 1H, OH), 6.62 (s, 1H, H7), 7.09 (s, 1H, H4), 7.27 (d, *J* = 1.8 Hz. 1H, H3), 8.85 (bs, 1H, NH); ^13^C-NMR (75.6 MHz, CDCl_3_): δ 23.4 (CO-Me), 50.8 (CO_2_Me), 70.1 (CH_2_), 92.8 (aryl C), 98.1 (C7), 107.5 (C4), 107.6 (C3), 122.1 (aryl C), 126.9 (C2), 138.7 (C5), 142.9 (C6), 161.6 (CO_2_Me), 204.9 (CO-Me); HRMS (+ESI): Found *m/z* 286.0684, [M + Na]^+^, C_13_H_13_NO_5_Na requires 286.0691.

*Methyl 5-hydroxy-6-((3-oxobutan-2-yl)oxy)-1H-indole-2-carboxylate* (**42**): The *title* compound was prepared as described in **GP-1** from methyl 5,6-dihydroxyindole-2-carboxylate (**18**) (100.4 mg, 0.485 mmol), 3-chloro-2-butanone (102.8 mg, 0.97 mmol), potassium carbonate (123.8 mg, 0.97 mmol) to give the product (43.0 mg, 32%) as a white solid; m.p. 198–200 °C; IR (KBr): *ν*_max_ 3379, 2987, 2943, 2343, 2107, 1946, 1694, 1618, 1523, 1439, 1379, 1294, 1238, 1144, 1069, 928, 857, 760, 726 cm^−1^; UV-vis (CH_3_CN): λ_max_ 209 nm (ε 40,300 cm^−1^ M^−1^), 319 (27,500); ^1^H-NMR (300 MHz, CDCl_3_): δ 1.45 (d, *J* = 2.1 Hz, 3H, CH-Me), 2.89 (s, 3H, C-Me), 3.87 (s, 3H, CO_2_Me), 4.02–4.09 (m, 1H, CH-Me), 5.61 (bs, 1H, OH), 6.97 (dd, *J* = 2.1, 0.9 Hz, 1H, H7), 7.03 (q, *J* = 1.3 Hz, 1H, H3), 7.04 (s, 1H, H4), 10.57 (bs, 1H, NH), ^13^C-NMR (75.6 MHz, CDCl_3_): δ 15.2 (CH-Me), 23.6 (CO-Me), 50.8 (CO_2_Me), 94.6 (CH-Me), 97.8 (C7), 107.2 (C4), 107.5 (C3), 121.9 (aryl C), 126.7 (C2), 133.5 (aryl C), 138.9 (C6), 144.0 (C5), 161.6 (CO_2_Me), 205.3 (CO-Me); HRMS (+ESI): Found *m/z* 300.0846, [M + Na]^+^, C_14_H_15_NO_5_Na requires 300.0842.

*Methyl 5-hydroxy-7,8-dimethyl-4-(2,2,2-trifluoroacetyl)-1H-furo[2,3-g]indole-2-carboxylate* (**43**): The *title* compound was prepared as described in **GP-2** from methyl 5-hydroxy-6-((3-oxobutan-2-yl)oxy)-1*H*-indole-2-carboxylate (**42**) (72.6 mg, 0.26 mmol) with 1:1 DCM:TFA (10 mL) to give the product (59.1 mg, 64%) as a yellow solid; m.p. 224–226 °C; IR (KBr): *ν*_max_ 3369, 2972, 2845, 2330, 2122, 1902, 1700, 1635, 1601, 1521, 1429, 1288, 1238, 1135, 1075, 927, 874, 790, 760 cm^−1^; UV-vis (CH_3_CN): λ_max_ 235 nm (ε 26,600 cm^−1^ M^−1^), 272 (23,100), 381 (31,000); ^1^H-NMR (300 MHz, CDCl_3_): δ 2.55 (d, *J* = 1.8 Hz, 3H, Me), 2.47 (d, *J* = 1.8 Hz, 3H, Me), 4.01 (s, 3H, CO_2_Me), 7.59 (t, *J* = 1.8 Hz, 1H, H3), 9.27 (bs, 1H, NH), 13.44 (bs, 1H, OH), ^13^C-NMR (75.6 MHz, CDCl_3_): δ 9.6 (C-Me), 12.3 (C-Me), 52.2 (CO_2_Me), 102.7 (C4), 109.7 (C3), 109.8, (C8), 110.7 (CF_3_) 118.6 (aryl C), 124.1 (C2), 126.3 (aryl C), 126.6 (aryl C), 140.8 (C5), 153.5 (aryl C), 157.7 (C7), 161.9 (CO_2_Me), 181.9 (CO-CF_3_); HRMS (+ESI): Found *m/z* 378.0568, [M + Na]^+^, C_16_H_12_F_3_NO_5_Na requires 378.0565.

### 3.2. Cell Biology Techniques

The SH-SY5Y and Kelly human neuroblastoma cell lines were generously donated by Dr. J. Biedler (Memorial Sloan-Kettering Cancer Center, New York, NY, USA). The MDA-MB-231 and MCF-7 breast cancer cell lines were purchased from the American Type Culture Collection. All cell lines were cultured under standard conditions at 37 °C in 5% CO_2_ as an adherent monolayer in Dulbecco’s modified Eagle’s medium supplemented with L-glutamine (DMEM) (Invitrogen, Waltham, MA, USA) and 10% foetal calf serum (FCS) (Thermo Fisher Scientific, Waltham, MA, USA).

### 3.3. Method for Cell Viability Assays

Cell viability was measured by the standard Alamar blue assay, as previously described [14]. Briefly, cells were allowed to attach for 24 h in 96-well culture plates. The cells were then continuously exposed to serial dilutions of the hydrazide-hydrazone derivatives for 72 h, either in the presence or absence of SAHA (0.5 or 1 µM), with five replicate wells for each determination. Cell viability was determined by the addition of 22 µL of Alamar blue reagent, recorded at comparative 0 h and 5 h values, using a Wallac 1420 Victor III spectrophotometer (GMI, Ramsey, MN, USA), which measured light absorbance in each well at 570 nm. The cell viability of each plate was calculated as a percentage compared to matched DMSO controls (0.5%). The mean (+/−SEM) is shown for three independent experiments.

### 3.4. Statistical Analysis

Results of the cell viability studies were statistically analysed using the two-tailed, unpaired Student’s *t*-test. Results are expressed as mean values with 95% confidence intervals.

## 4. Conclusions

In this manuscript, methyl 4,6-dihydroxyindole (**17**), 5,6-dihydroxyindole (**18**), 5-hydroxy-6-methoxyindoles-2-carboxylate (**19**) and 6-hydroxy-5-methoxy-2-carboxylate (**20**) were used to generate new classes of tetracyclic and tricyclic furoindole systems. Tricyclic furo[3,2-*e*]indoles and furo[2,3-*g*]indoles were readily prepared by the treatment of the corresponding monohydroxyindoles **19** and **20** with α-haloketones followed by the cyclization with a catalytic amount of acid. Methyl 4,6-dihydroxyindole-2-carboxylate **17** afforded the di-alkylated systems as the only products upon alkylation, while in the case of methyl 5,6-dihydroxyindole-2-carboxylate **18**, the hydroxyl group at the C6 was more reactive than the one at C5, generating the *O*-C6 monoalkylated intermediates as minor products, with higher yields of di-alkylated intermediates also formed. However, while the desired tetracyclic furoindoles could be generated by the treatment of intermediates derived from 4,6-dihydroxyindole **17**, attempts failed to produce the desired tricyclic and tetracyclic products from the 5,6-dihydroxyindole **18** system. Presumably the cyclization reaction of **42** gives the intermediate benzofuran product which is able to generate the unexpected tricyclic furoindole **43** containing a trifluoroacetyl group at the C4 position.

Biological studies revealed that the furoindole systems showed moderate cytotoxicity towards neuroblastoma and breast cancer cells. However, compound **43** was the only product which could be considered as a promising SAHA enhancer with 29% additional reduction of SH-SY5Y viability compared to SAHA alone. It was also interesting to note that the non-cyclized intermediate compound **30** displayed a strong cytotoxicity with IC_50_ values of 13.3 and 3.1 µM against the SH-SY5Y and Kelly neuroblastoma cell lines, and was even more potent than its corresponding mono-cyclized (**34**) and di-cyclized (**32**) analogues.

## Figures and Tables

**Table 1:** Activity of compounds **30**, **32** and **34** against neurobalstoma cancer cell lines.

Compound	IC_50_ (µM) ^a^ SH-SY5Y	KELLY
**30**	13.3	3.1
**32**	>20	>20
**34**	>20	>20

^a^ Compound concentration required to reduce cell viability by 50%, as determined by the Alamar Blue assay, under conditions allowing untreated controls to undergo at least five consecutive rounds of multiplication.

## Data Availability

Data are contained within the article and the figures in Supporting materials.

## Sample Availability

Samples of the compounds are available from the authors.
